# Social alienation and influencing factors among caregivers of stroke patients in China: a cross-sectional study

**DOI:** 10.3389/fpsyt.2025.1544692

**Published:** 2025-06-05

**Authors:** Lang Xu, Shanshan Liu, Mao Luo, Min Li, Cong Wang

**Affiliations:** ^1^ Department of Neurosurgery, West China Hospital, Sichuan University/West China School of Nursing, Sichuan University, Chengdu, China; ^2^ Evidence-based Nursing Center, West China Hospital, Sichuan University/West China School of Nursing, Sichuan University, Chengdu, China; ^3^ Department of Nursing, The Affiliated Ganmei Hospital of Kunming Medical University, Kunming, China; ^4^ Department of Radiology, West China Hospital, Sichuan University, Chengdu, China

**Keywords:** stroke, caregivers, social alienation, care burden, social support, loneliness

## Abstract

**Introduction:**

As the primary bearers of post-stroke rehabilitation and long-term care, caregivers of stroke patients in China face a profound sense of social alienation that has not yet been fully recognized due to issues such as role-related limitations. Especially in the context of China’s family caregiving model and evolving social support system, this sense of alienation not only undermines the physical and mental health of caregivers, but also ultimately affects the quality of ongoing rehabilitation support. This is undoubtedly detrimental to both care providers and recipients. Therefore, the aim of this study was to investigate the level of social alienation and its influencing factors among Chinese stroke caregivers.

**Methods:**

Participants for this study were selected from caregivers of stroke patients who visited the neurology department of a comprehensive hospital in Kunming between February and August 2023. A total of 222 stroke caregivers were assessed using the General Alienation Scale, the Zarit Caregiver Burden Interview, the Social Support Rating Scale, and the University of California at Los Angeles-Loneliness Scale.

**Results:**

The mean social alienation scores for the stroke caregivers included in this study were 40.45 ± 4.76 (range:24-51).Multivariate linear regression analysis revealed that the marital status of caregivers, whether they live with the patient, their knowledge of the disease, social support, caregiving burden, and loneliness are significant factors influencing social alienation in stroke caregivers (all *p* < 0.05).

**Conclusions:**

Caregivers of stroke patients experience high levels of social alienation. It is crucial to address the social alienation of these caregivers, particularly those who live with the patient, are divorced or widowed, have limited knowledge of the disease, bear a heavy caregiving burden, have low levels of social support, and experience high levels of loneliness. Targeted and individualized interventions should be developed to reduce their social alienation.

## 1 Introduction

Stroke is a leading cause of adult death and disability in China and other parts of the world (1). Globally, there are about 12.2 million new cases of stroke each year (2). At the same time, with the accelerating aging of the population, the number of current stroke patients in China is the highest in the world, and the incidence rate is increasing year by year, with the burden of disease becoming heavier and heavier (3). Relevant data show that 50%-80% of stroke survivors suffer from varying degrees of cognitive, speech, or somato-motor dysfunction (4), and severely impacting their daily functioning. Worse still, caregiving tasks for more than 90% of stroke patients are often provided directly by untrained and ill-prepared family members due to a variety of cultural, economic, and uneven distribution of healthcare resources (5).In China, under the influence of traditional filial piety culture (6), caring for sick parents is regarded as an unshakable responsibility of children, a concept that makes children, when faced with stroke patients, often take the initiative to undertake the task of caring for them and insist on fulfilling their obligations even when faced with financial pressure or obstacles to their career development, thus adding to the overall burden. Huan et al. (7) found that more than 55% of stroke caregivers had a moderately-severe burden of caregiving in a cross-sectional survey of 286 stroke caregivers. In addition, it has been noted that about 34%-52% of stroke caregivers (8) report mental health issues such as anxiety and depression, and the physical and mental health of stroke caregivers is facing great challenges.

In recent years, an interaction has been found between caregiving burden and social alienation, such that the caregiver social alienation level is inextricably linked to caregiving burden (9). This interaction not only affects the mental health of stroke caregivers, but may also lead to a range of adverse clinical and life outcomes (10), which in turn further exacerbate their burden and level of social alienation. Within the family, social alienation of stroke caregivers may lead to tensions within the family (11), and even trigger inadequate social support for families of stroke patients (12), affecting the patient’s overall recovery process and social integration. Stroke caregivers are significant to the healing process of patients, but are at an increasing risk for experiencing adverse effects. Due to the complexity of the disease and the long recovery period, caregiver support is indispensable for the healing of stroke patients, which results in caregivers having to give up social activities such as exercise (13), travel (14), potentially lack of time, energy and financial support for social contact, and even active avoidance of socialization, which aggravates caregivers’ social alienation (14).

Social alienation is a phenomenon in which the individual lacks contact with others during social activities, resulting in a failure to adequately satisfy social desires, which in turn produces negative emotions such as loneliness and helplessness, as well as negative behaviors such as refusing to socialize (15). Social alienation is not exclusive to patients, rather it appears more prominent in the caregiver community. A systematic evaluation (16) summarized that the prevalence of social alienation among caregivers of people with dementia was approximately 37.1%. In Australia, caregivers of people with schizophrenia (17) have a risk of social alienation 10 times greater than community caregivers(e.g., community nurses). Nowadays, the adverse consequences brought about by the social alienation of caregivers have increasingly drawn people’s attention and emphasis. A study by Hayes et al. (17) showed that more than 40% of caregivers suffering from social alienation may have anxiety or depression. In addition, male caregivers are four times more likely to be at risk for social alienation than women (18). Not only does social alienation substantially increase the risk of multiple chronic illnesses in caregivers (19), but also leads to other psychiatric disorders that diminish the ability to care and reduce the quality of care (20). Studies of stroke patients and their caregivers have also shown that the presence of social alienation is associated with stroke risk and a 29% higher mortality rate (21), which renders stroke caregivers unfavorable for performing medical tasks and even more unfavorable for recovery of stroke patients.

The sudden nature of stroke and its potential for significant functional impairment in patients require caregivers to provide high levels of care. In this context, caregivers are often confronted with the adverse effects of their caregiving role. However, much of the current research on stroke caregivers focuses on caregiving burden, negative psychological emotions, and little attention has been paid to their social alienation. Little is known about studies on social alienation in stroke caregivers, forcing us to revisit the assessment and anticipation of social alienation in stroke caregivers. In view of this, the main purpose of this study was to investigate the occurrence of social alienation in caregivers of stroke patients and to analyze and explore the possible influencing factors, which would serve as a reference for the subsequent development of relevant interventions.

## 2 Methods

### 2.1 Design and participants

This study employed a convenience sampling method using a cross-sectional study design. The participants were caregivers of stroke inpatients, recruited from the neurology department of a general hospital in Kunming between February 2023 and August 2023. The inclusion criteria for patients were as follows: (a) meeting the diagnostic criteria for stroke as defined in the literature (22), confirmed by cranial CT or MRI; (b) age ≥18 years; and (c) stroke diagnosis confirmed ≥7 days prior to participation. Patients with other acute critical illnesses or malignant tumors were excluded.

Caregivers were included based on the following criteria: (a) being a relative of the patient(e.g., blood relatives, marital relatives), providing care without financial compensation; (b) age ≥18 years; (c) providing care for at least 4 hours per day; and (d) possessing independent reading and writing abilities. Exclusion criteria for caregivers included: (a) experienced other major stressful events in the last three months (e.g., serious car accident, bereavement or job loss); and (b) having mental or physical disabilities that would prevent completion of the survey(e.g., blindness, schizophrenia).

Upon obtaining informed consent from both patients and caregivers, a total of 230 caregivers participated in the study. Of these, 222 caregivers completed the questionnaire in a valid and thorough manner, resulting in an effective response rate of 96.5%. This study was approved by the hospital’s ethics committee.

### 2.2 Questionnaire

#### 2.2.1 Sociodemographic data

Based on a thorough literature review and expert consultations, our research team developed a general information questionnaire. The questionnaire includes details such as the patient’s age, gender, and stroke classification, as well as the caregiver’s age, gender, marital status, relationship to the patient, and knowledge of the patient’s disease.

#### 2.2.2 General alienation scale

The scale was developed in 1977 by Jessor et al. (23) to assess individuals’ feelings of isolation and uncertainty about participating in activities. It primarily measures four dimensions: self-alienation, social alienation, powerlessness, and meaninglessness. The scale consists of 15 items with a total possible score of 60, where higher scores indicate a greater level of social alienation. A 4-point Likert scale is used, with response options ranging from 1 (strongly disagree) to 4 (strongly agree). The Cronbach’s alpha coefficient of the scale is 0.81, the split-half reliability is 0.80, and the test-retest reliability is 0.76.The Chinese version of the GAS demonstrated good reliability (Cronbach’s α = 0.77) (24). In addition, this scale has been widely used in Chinese stroke patients and has been shown to have good reliability.

#### 2.2.3 Zarit caregiver burden interview

The Chinese version of the scale was revised by Wang Lie et al. in 2006 (25), which includes two subscales: personal burden (12 items) and burden of responsibility (6 items).The scale utilizes a 5-point Likert scoring system, where 0 indicates “never” and 4 indicates “always.” The total score ranges from 0 to 88, with higher scores reflecting greater caregiving burden. A total score of less than 21 indicates no or mild burden, 21 to 40 indicates moderate burden, and a score above 40 indicates heavy burden. The Cronbach’s alpha coefficient for the scale is 0.823, demonstrating good reliability and validity.

#### 2.2.4 Social support rating scale

The scale, developed by Xiao Shuiyuan (26), assesses three dimensions of social support: subjective support, objective support, and the degree of social support utilization. The scale consists of 10 items, of which items 1–4 and 8–10 are positively scored on a Likert 4 scale (1–4), item 5 is scored as a single item (none is 1, rarely 2, generally 3, and fully supported 4), and “how many sources” is scored for items 6 and 7, for a total of 66 points. Higher scores indicate a greater level of social support. A total score of less than 23 suggests a low level of social support, a score between 23 and 45 indicates a moderate level, and a score between 45 and 66 reflects a high level of social support. The scale demonstrates good internal consistency, with a Cronbach’s alpha coefficient of 0.90.

#### 2.2.5 University of California at Los Angeles-Loneliness Scale

The UCLA Loneliness Scale, developed by psychologist Russell and colleagues in 1978 (27), is designed to assess the discrepancy between an individual’s desire for social interaction and their actual level of social engagement, which contributes to feelings of loneliness. The scale consists of 20 items rated on a 4-point Likert scale, with responses ranging from 1 (“never”) to 4 (“almost every day”). The total score ranges from 20 to 80 points, where a score of 20 or below indicates no loneliness, 21–40 indicates mild loneliness, 41–60 indicates moderate loneliness, and 61–80 indicates severe loneliness. Higher scores reflect greater levels of loneliness. The Chinese version of the UCLA Loneliness Scale has demonstrated good reliability and validity, with a Cronbach’s alpha coefficient of 0.887.

### 2.3 Sample size

In this study, we used the sample size estimation formula for cross-sectional surveys to estimate our required sample size (28):


n =  Z2α2(1−p)pδ2

In the formula, Z(α/2) is set to 1.96 (for a two-tailed test with α=0.05). The prevalence *p* is based on the reported rates of social alienation among caregivers from international literature, which range from 30% to 45%. The margin of error δ is set to 5%. Anticipating a 20% rate of invalid questionnaires, the calculated sample size ranges from 197 to 232 participants. After considering these factors, the final sample size was determined to be 230 participants. A total of 230 questionnaires were distributed for this study, and 230 were returned. After screening, 8 questionnaires were excluded due to a high number of missing items or identical responses across 10 consecutive items. Consequently, the final sample consisted of 222 valid questionnaires.

### 2.4 Data collection

In this study, data collection was done through face-to-face paper questionnaire completion. During the study period (February to August 2023), Kunming was released from epidemic control and no epidemic-related disturbances were reported, so this study was less affected by the COVID-19 pandemic. During the survey process, the researchers explained the purpose, significance, and specific content of the study to the caregivers. Caregivers were asked to complete the questionnaire independently. In cases where reading or comprehension difficulties arose, the researchers provided detailed explanations for each item until the caregivers fully understood before proceeding. Each questionnaire took no more than 30 minutes to complete. Upon completion, all questionnaires were collected on-site and immediately checked for completeness and validity. To ensure data accuracy, the questionnaires were reviewed by two nursing graduate students. Once verified, the data were entered into Excel 2019 on the same day.

### 2.5 Statistical methods

The data were imported from Excel into SPSS 25.0 for statistical analysis. All data underwent normality and homogeneity of variance tests. Descriptive statistics, including frequencies and percentages, were used to summarize the demographic characteristics of the study participants. Means ± standard deviations(M ± SD)were used to describe the scores of the various study variables. Independent samples t-tests or one-way ANOVA were employed to compare differences in social alienation scores among the study participants. Pearson correlation analysis was conducted to examine the relationships between caregiver social support, caregiving burden, loneliness, and social alienation. Finally, multiple linear regression analysis was performed to identify the factors influencing the level of social alienation in caregivers of stroke patients. A *p*-value of less than 0.05 was considered statistically significant.

## 3 Results

### 3.1 Sociodemographic characteristics of stroke caregivers

A total of 222 (n=222) stroke caregivers were enrolled in this study. The majority of caregivers were aged 31 to 50 years (n=95, 42.8%), while the fewest were aged 71 years or older (n=10, 4.5%). Among the caregivers, n=85(38.3%) were male and n=137 (61.7%) were female. Additionally,n=140 (63.1%) caregivers had co-caregivers, while n=82 (36.9%) caregivers provided care individually (**Table 1**).

**Table 1:** Sociodemographic characteristics of stroke caregivers and outcome of univariate analysis (*N=222)*.

Variables		Frequency (%)	GAS mean score	*t/F*	*p*
Age (years)	18∼30	27 (12.2)	39.85 ± 4.23	3.751	**0.012**
	31∼50	95 (42.8)	39.42 ± 5.04		
	51∼70	90 (40.5)	41.64 ± 4.31		
	≥71	10 (4.5)	41.30 ± 5.25		
Gender	Male	85 (38.3)	40.16 ± 4.05	−0.726	0.469
	Female	137 (61.7)	40.64 ± 5.15		
Ethnic group	Han ethnic group	201 (90.5)	40.50 ± 4.69	0.464	0.643
	Non-Han ethnic group	21 (9.5)	40.00 ± 5.43		
Educational level	Primary school or lower	25 (11.3)	43.20 ± 4.28	13.621	**<0.001**
	Junior high school	73 (32.9)	41.31 ± 4.40		
	Senior high school	69 (31.1)	41.02 ± 3.98		
	University or higher	55 (24.8)	37.36 ± 4.91		
Marital status	Single	20 (9.0)	39.30 ± 4.74	4.833	**0.003**
	Married	191 (86.0)	40.29 ± 4.71		
	Widowed	4 (1.8)	44.50 ± 3.00		
	Divorced	7 (3.2)	46.00 ± 2.08		
Employment status	Unemployed	14 (6.3)	43.14 ± 5.85	4.523	**0.004**
	Employed	73 (32.9)	39.02 ± 4.71		
	Retired	52 (23.4)	40.61 ± 4.57		
	Others	83 (37.4)	41.16 ± 4.41		
Monthly income (yuan/person)	<3000	69 (31.1)	42.71 ± 3.98	15.143	**<0.001**
	3000˜5000	89 (40.1)	40.13 ± 4.45		
	>5000	64 (28.8)	38.48 ± 4.99		
Chronic disease	No	157 (70.7)	39.82 ± 4.90	−3.132	**0.002**
	Yes	65 (29.3)	41.98 ± 4.04		
Daily care hours (h)	≤8	44 (19.8)	38.75 ± 4.97	4.216	**0.016**
	8˜16	65 (29.3)	40.38 ± 3.75		
	16˜24	113 (50.9)	41.16 ± 5.05		
Co-caregivers	Yes	140 (63.1)	39.64 ± 4.51	−3.419	**0.001**
	No	82 (36.9)	41.85 ± 4.87		
Total care time (month)	<1	135 (60.8)	39.58 ± 4.63	6.902	**0.001**
	1˜3	26 (11.7)	42.76 ± 4.17		
	≥3	61 (27.5)	41.40 ± 4.82		
Knowledge of the patient’s disease	definite	41 (18.5)	36.95 ± 4.99	20.259	**<0.001**
	Partially definite	163 (73.4)	40.93 ± 4.29		
	indefinite	18 (8.1)	44.11 ± 3.73		
Relationship with the patients	spouse	79 (35.6)	41.39 ± 5.01	3.576	**0.015**
	Children or their spouses	124 (55.9)	39.69 ± 4.52		
	parent	3 (1.3)	46.00 ± 1.73		
	Others	16 (7.2)	40.75 ± 4.40		
Living with the patients	Yes	147 (66.2)	41.08 ± 4.68	2.767	**0.006**
	No	75 (33.8)	39.24 ± 4.70		
Place of residence	Urban area	132 (59.5)	39.78 ± 4.59	−2.608	**0.010**
	Rural area	90 (40.5)	41.45 ± 4.84		

Post-hoc Tukey tests revealed significant differences between: Divorced vs. Married (p=0.001), Indefinite vs. Definite disease knowledge (p<0.001). Bold values are statistically significant.

### 3.2 Univariate analysis

Further analysis revealed that the general demographic characteristics of stroke patients did not significantly differ in terms of social alienation scores (*p* > 0.05). However, significant differences in social alienation scores were observed based on the following caregiver characteristics: age, educational level, marital status, employment status, monthly family income, presence of chronic diseases, daily caregiving hours, presence of co-caregivers, total caregiving duration, knowledge of the disease, relationship with the patient, cohabitation with the patient, and place of residence (*p* < 0.05). Detailed data are presented in **Table 1**.

### 3.3 Current analysis of social alienation, caregiving burden, social support and loneliness among stroke caregivers

The results of this study showed that the social alienation score of caregivers of stroke patients ranged from 24 to 51 with a total score of (40.45 ± 4.76). The dimensional scores were in the order of other alienation (12.28 ± 2.42), self-alienation (8.36 ± 1.77), skepticism (10.18 ± 2.09), and meaninglessness (9.62 ± 1.81). Caregiving burden scores ranged from 6 to 76 with a total score of (33.66 ± 11.56), social support scores ranged from 18 to 56 with a total score of (50.77 ± 5.91), and loneliness scores ranged from 28 to 65 with a total score of (50.77 ± 5.91) (**Table 2**).

**Table 2:** Scores of social alienation in stroke caregivers *(N=222)*.

Items	Range	Mean score( $\bar{X}\pm S$ )
Total score for social alienation	24˜51	40.45 ± 4.76
Total score for care burden	6˜76	33.66 ± 11.56
Total score for social support	18˜56	35.96 ± 7.28
Total score for loneliness	28˜65	50.77 ± 5.91

### 3.4 Correlation analysis

Pearson correlation analysis revealed that social alienation in caregivers was positively correlated with caregiving burden (*r* = 0.452, *p* < 0.01) and loneliness (*r* = 0.434, *p* < 0.01), and negatively correlated with social support (*r* = -0.491, *p* < 0.01). Specifically, higher levels of caregiving burden and loneliness were associated with higher levels of social alienation, while higher levels of social support were associated with lower levels of social alienation in caregivers of stroke patients (**Table 3**).

**Table 3:** Correlation analysis of social alienation,care burden, social support, and loneliness in stroke caregivers (R-values).

	Social alienation	Care burden	Social support	Loneliness
Social alienation	1			
Care burden	0.452**	1		
Social support	−0.491**	−0.458**	1	
Loneliness	0.434**	0.322**	−0.241**	1

** indicates a significant correlation at the 0.01 level (two-tailed).

### 3.5 Multiple linear regression analysis

The results of multiple linear regression analysis showed that the model fit was good, and the variance inflation factor (VIF) of the model was less than 5, between 1.141 and 1.952, suggesting that there was no problem of covariance, in which the caregiver’s marital status, knowledge of the patient’s disease, whether they lived with the patient, the caregiving burden, social support, and loneliness were the six variables had an effect on social alienation, and the cumulative explanation of social alienation by the explainable independent variables was 47.9%. Detailed data are shown in **Table 4**.

**Table 4:** Multivariate analysis of social alienation in stroke caregivers *(N=222)*.

Variables	Control variables	B	SE	β´	t	p
Constant		29.84	3.936		7.582	<0.001
Marital status	Single	Ref	Ref	Ref	Ref	Ref
Married		0.993	1.091	0.072	0.910	0.364
Widowed		5.200	2.542	0.146	2.045	0.042*
Divorced		6.700	2.038	0.246	3.287	0.001*
Knowledge of the patient’s disease	definite	Ref	Ref	Ref	Ref	Ref
Partially definite		3.987	0.768	0.371	5.195	<0.001*
indefinite		7.160	1.242	0.411	5.764	<0.001*
Living with the patients	Yes	Ref	Ref	Ref	Ref	Ref
No		-1.842	0.666	-0.183	-2.767	0.006*
Care burden		0.069	0.025	0.167	2.749	0.007*
Social support		-0.141	0.04	-0.216	-3.529	0.001*
Loneliness		0.241	0.042	0.3	5.693	<0.001*

* indicates a significant correlation at the 0.05 level, R²=0.517, Adjusted R²=0.479, F=13.710, p<0.001.

## 4 Discussion

This study provides the first evidence of social alienation among caregivers of stroke patients in China. The mean total score for social alienation was 40.45 ± 4.76, indicating a high level of social alienation among these caregivers. At the same time, this finding is a step closer to confirming that caregivers, like stroke patients (29), are a group affected by social alienation, and that the problem of social alienation in stroke caregivers should not be ignored. Further analysis suggests that the high levels of social alienation among caregivers may be attributed to several factors. Stroke often occurs suddenly and without warning, leaving caregivers unprepared for the caregiving role. The demanding nature of caregiving can exacerbate their physical and mental health, leading to increased psychological burdens and behaviors such as loneliness and avoidance. Additionally, the decline in self-care abilities of stroke patients often forces caregivers to abandon work, social gatherings, and travel, thereby reducing their social interactions and opportunities. This social disengagement leads to social alienation, which, in turn, increases caregiving burden and contributes to negative psychological outcomes (14, 30). In addition it has been noted that this is also associated with inadequate social support for caregivers in the caregiving process (31). This is because it limits the caregiver’s access to emotional and financial resources, which leads to higher levels of social alienation. Therefore, healthcare providers should prioritize addressing the social alienation of caregivers of stroke patients. Strategies to improve social support, such as leveraging social networks (32),should be implemented. Additionally, providing psychological counseling services to help caregivers develop a positive disease perspective can reduce their caregiving burden and enhance both the quality of care and their own well-being, ultimately lowering their levels of social alienation.

In our study, another important finding is that the marital status of caregivers, their knowledge of the patient’s disease, whether they live with the patient, the caregiving burden, social support, and loneliness are significant factors influencing social alienation among caregivers. The results show that caregivers who are divorced or widowed experience higher levels of social alienation, consistent with findings by Wang Shuo et al. (33). The underlying reasons for this may include the limited emotional and financial support available to divorced or widowed caregivers due to changes in family dynamics. When a patient experiences a sudden stroke, the lack of support can lead to increased economic pressure and caregiving burden, resulting in heightened psychological stress. Without adequate outlets for this stress, caregivers may resort to avoidance behaviors, withdrawing from reality and social interactions, which can lead to self-isolation and increased social alienation (11, 34). These findings suggest that healthcare providers should pay particular attention to the psychological state of divorced or widowed caregivers in clinical practice. Targeted psychological interventions should be provided to help these caregivers manage their stress, encourage social engagement, and improve their self-care and health management.

Additionally, our study found that caregivers who have less knowledge about stroke and the patient’s condition experience higher levels of social alienation, a result consistent with the findings of Kobayashi (35). This may be related to the caregivers’ understanding of the disease. The recovery process for stroke patients often requires active participation and support from caregivers. Accurate knowledge about stroke can enhance the caregivers’ ability to assist in the patient’s rehabilitation and increase their motivation for treatment. Therefore, it is essential to improve caregivers’ understanding of stroke through various channels and methods, including the use of new media technologies. Educating both patients and caregivers about the disease can help them develop a more accurate and positive perspective, which is beneficial for the patient’s recovery and overall well-being.

The study results indicate that cohabiting with stroke patients also influences caregivers’ social alienation. We found that caregivers who live with stroke patients experience higher levels of social alienation. Influenced by traditional Chinese filial piety, the responsibility for post-discharge rehabilitation and ongoing care of stroke patients often falls on family members, such as children or parents. Due to the lengthy recovery period and the demanding nature of caregiving (36), caregivers are often compelled to live with the patient to provide better care. This arrangement forces them to reduce their work and social interactions (37), leading their lives to revolve around the needs of the stroke patient. Over time, this can result in deteriorated physical and mental health, strained caregiving relationships, and increased social alienation. These findings highlight the need for healthcare providers to delve into the inner world of caregivers, establish trusting relationships, and encourage them to share their caregiving experiences and pressures. Strengthening communication and maintaining regular contact with caregivers can help address their needs and provide necessary support.

The caregiving burden imposed by stroke is prolonged and multifaceted, affecting caregivers physically, psychologically, and economically. Our study found a positive correlation between caregiving burden and social alienation among caregivers. Similarly, Ross et al. (9) have identified a bidirectional relationship between social alienation and caregiver burden. Previous research has shown that caregiving burden is a strong predictor of social alienation in caregivers (38). Stroke patients, due to the impact of the disease, often lose or partially lose their ability to perform daily activities, becoming dependent on caregivers. This dependency limits the caregivers’ ability to engage in social activities and maintain social connections, leading to negative emotions and increased social alienation (39). Therefore, healthcare providers should actively work to improve the negative caregiving experiences of stroke caregivers. Interventions such as psychoeducation and cognitive-behavioral therapy (40) can help reduce their psychological burden. Additionally, at the societal level, efforts should be made to enhance public awareness of stroke and increase support, thereby alleviating the economic burden on caregivers.

This study also confirms the association between social alienation and social support, showing that caregivers with lower levels of social support experience higher levels of social alienation, consistent with the findings of Liang Yaqing et al. (41). Under the pressure of caregiving, stroke caregivers often seek emotional support from their families and economic support from external sources (42). They also desire to maintain good social relationships. However, the demands of caregiving responsibilities and other care needs often limit the amount of social support they receive, leading to increased social alienation (43). Therefore, it is crucial to establish robust social support systems to enhance the level of social support for stroke caregivers. Encouraging social interactions and promoting social integration can help mitigate the effects of social alienation and improve the overall well-being of caregivers.

Notably, our study found that caregivers with higher levels of loneliness experience higher levels of social alienation, consistent with previous research findings (44). This may be due to the numerous stressors that stroke caregivers face during the caregiving process, which can lead to social alienation and feelings of loneliness. While previous studies often view loneliness as a subjective aspect of social alienation, it is important to recognize that loneliness and social alienation are related but distinct concepts. Both refer to unmet social needs, but loneliness emphasizes the quality of an individual’s social network (45, 46). Research has found that acceptance and commitment therapy (47) could be a potential option for reducing loneliness. Because it could help individuals learn to live in harmony with negative internal experiences. During the caregiving process, stroke caregivers often experience reduced social interactions and unmet social needs, leading to a loss of confidence in the effectiveness of rehabilitation treatments. This can result in feelings of powerlessness, helplessness, and despair, further exacerbating their sense of loneliness and social alienation. These findings suggest that healthcare providers can help reduce the loneliness levels of stroke caregivers through interventions such as mindfulness meditation (48), music therapy, and peer support. By addressing loneliness, these methods can also help lower the levels of social alienation experienced by caregivers.

## 5 Limitation

There are several limitations that need to be acknowledged in this study. First, due to constraints in time and human resources, our sample was limited to caregivers of stroke patients from a tertiary hospital in Kunming. This may result in a smaller and less representative sample. Future research should aim to conduct multi-center, large-scale studies across other regions in China. Secondly, the use of the social alienation scale in the caregiver population is not yet widespread, which may limit its accuracy in reflecting the social alienation experienced by caregivers. Future research should consider the cultural diversity and differences to develop more targeted and culturally appropriate social alienation scales for caregivers of different patient populations. In addition, cross-sectional studies alone may not be able to reveal the real reasons and experiences behind the social detachment of stroke caregivers, and in the future, qualitative studies may be conducted to explore in depth the issue of social detachment among caregivers of stroke patients in China. Finally, our study focused solely on caregivers of hospitalized stroke patients. Given the long-term nature of stroke rehabilitation, home and community settings are also primary environments for caregiving. Future research should incorporate these factors and conduct longitudinal studies to assess the dynamic changes in social alienation over time.

## 6 Conclusion

The results of this study indicate that caregivers of stroke patients experience high levels of social alienation, which is influenced by the caregiver’s marital status, cohabitation with the patient, knowledge of the disease, social support, caregiving burden, and loneliness. It is crucial for relevant authorities to recognize the significant role and psychological well-being of caregivers while focusing on the needs of stroke patients. Efforts should be made to encourage social interactions among caregivers, enhance their social support, and reduce feelings of loneliness. Additionally, establishing a robust social support system to alleviate the caregiving burden can help mitigate social alienation among caregivers. Future research should extend to caregivers of other types of illnesses and develop targeted interventions to address the unique challenges faced by these caregivers.

## Data Availability

The original contributions presented in the study are included in the article/supplementary material. Further inquiries can be directed to the corresponding authors.
